# Surgical Downstaging in an Open-Label Phase II Trial of Denosumab in Patients with Giant Cell Tumor of Bone

**DOI:** 10.1245/s10434-015-4634-9

**Published:** 2015-06-02

**Authors:** Piotr Rutkowski, Stefano Ferrari, Robert J. Grimer, Paul D. Stalley, Sander P. D. Dijkstra, Andrzej Pienkowski, Gualter Vaz, Jay S. Wunder, Leanne L. Seeger, Amy Feng, Zachary J. Roberts, Bruce A. Bach

**Affiliations:** Department of Soft Tissue/Bone Sarcoma and Melanoma, Maria Sklodowska-Curie Memorial Center and Institute of Oncology, Roentgen Str 5, 02-781 Warsaw, Poland; Chemotherapy Unit, Istituto Ortopedico Rizzoli, Bologna, Italy; Orthopaedic Oncology Unit, Royal Orthopaedic Hospital, Birmingham, UK; Department of Orthopaedic Surgery, Royal Prince Alfred Hospital, Sydney, NSW Australia; Department of Orthopedic Surgery, Leiden University Medical Center, Leiden, The Netherlands; Department of Surgery, Centre Leon Berard, Lyon, France; Samuel Lunenfeld Research Institute, Mount Sinai Hospital, University of Toronto, Toronto, ON Canada; Department of Radiology, David Geffen School of Medicine at University of California Los Angeles, Los Angeles, CA USA; Global Biostatistical Science, Amgen Inc., Thousand Oaks, CA USA; Global Development Oncology Therapeutics, Amgen Inc., Thousand Oaks, CA USA

## Abstract

**Background:**

Surgical resection with curative intent for giant cell tumor of bone (GCTB) may be associated with severe morbidity. This interim analysis evaluated reduction in surgical invasiveness after denosumab treatment in patients with resectable GCTB.

**Methods:**

Patients with primary or recurrent GCTB, for whom the initially planned surgery was associated with functional compromise or morbidity, received denosumab 120 mg subcutaneously every 4 weeks (additional doses on days 8 and 15 of the first cycle). Planned and actual GCTB-related surgical procedures before and after denosumab treatment were reported. Patients were followed for surgical outcome, adverse events, and recurrence following resection.

**Results:**

Overall, 222 patients were evaluable for surgical downstaging (54 % were women; median age 34 years). Lesions (67 % primary and 33 % recurrent) were located in the axial (15 %) and appendicular skeleton (85 %). At the data cutoff date, most patients had not yet undergone surgery (*n* = 106; 48 %) or had a less morbid procedure (*n* = 84; 38 %) than originally planned. Median (interquartile range) time on denosumab was 19.5 (12.4–28.6) months for the 106 patients who had not undergone surgery and were continuing on monthly denosumab. Native joint preservation was 96 % (*n* = 24/25) for patients with planned joint/prosthesis replacement and 86 % (*n* = 30/35) for patients with planned joint resection/fusion. Of the 116 patients who had surgery (median postsurgical follow-up 13.0 [8.5–17.9] months), local recurrence occurred in 17 (15 %) patients.

**Conclusion:**

For patients with resectable GCTB, neoadjuvant denosumab therapy resulted in beneficial surgical downstaging, including either no surgery or a less morbid surgical procedure.

**Electronic supplementary material:**

The online version of this article (doi:10.1245/s10434-015-4634-9) contains supplementary material, which is available to authorized users.

Giant cell tumor of bone (GCTB) is an aggressive, bone lytic, osteoclastogenic stromal tumor that mainly occurs in young adults.^1^,^2^ It commonly presents as an epiphyseal, monostotic lytic lesion most often found in the distal femur, proximal tibia, and distal radius.^1^ It is characterized by progressive growth and geographic bone lysis, leading to cortical bone expansion or dissolution with or without soft tissue extension. Symptoms generally include pain, swelling, and impaired mobility and function.^1^ Local mechanical load and joint function compromise are common in untreated disease. Rarely, GCTB can undergo malignant transformation. In addition, 1–4 % of GCTB cases give rise to pulmonary metastases even when the histologic appearance remains benign.^3^

Currently, surgical removal of the lesion remains the only curative intent treatment for GCTB;^4^ however, local recurrence or metastasis can still occur following curative intent surgery with modern imaging and high-speed burring.^5^,^6^ The most common form of surgical treatment for GCTB is aggressive local curettage with or without packing of the defect with bone cement or bone graft and internal fixation. The aim of this approach is to remove the tumor while preserving the local functional anatomy, including the articular joint surface. Varying rates of local recurrence have been reported after intralesional surgical therapy, and have led to the suggestion that the use of local adjuvants such as phenol, peroxide, water, or liquid nitrogen may further improve local control.^7^–^11^ More aggressive surgical approaches employing wide resection of the involved bone may be chosen to achieve tumor removal and potentially decrease the risk of local recurrence, at the cost of greater functional compromise.^7^ Major excision and resection of the involved bone (e.g. amputation, joint resection, or hemipelvectomy) for advanced GCTB,^3^ even if some form of bone or joint reconstruction is possible, is associated with significant functional deficit or morbidity.^12^

Denosumab, a monoclonal antibody directed against the receptor activator of nuclear factor-kappa β ligand (RANKL), has recently been approved in the United States, Europe, and Japan for the treatment of adults and skeletally mature adolescents with GCTB that is unresectable or when surgical resection is likely to result in severe morbidity.^13^–^15^ GCTB has been shown to be pathogenetically driven by pervasive expression of osteoclastic differentiation signals by tumor mononuclear stromal cells.^16–19^ Immunohistochemical and molecular probes have shown that stromal cell elements of GCTB strongly produce and express RANKL.^17^ RANKL appears to play an autocrine role in lesion development fostering and maintaining osteoclast formation, activation, and survival,^18^ resulting in continuous bone resorption^19^,^20^ via activating RANK receptor-positive osteoclast-like giant cells and their precursors.^16^,^21^

Previous results from an open-label, single-arm, phase II study demonstrated sustained denosumab-induced tumor responses in patients with GCTB (based on assessment of histologic or radiologic response).^22^ Denosumab treatment produced rapid and substantial suppression of bone turnover and significant reduction in the numbers of multinucleated giant cells seen in post-treatment resection specimens, as well as a marked reduction in the number and cross-sectional area of residual mononuclear stromal cells.^17^,^22^ There was a consistent finding of complete or near complete elimination of RANKL-producing stromal cells and disappearance of original RANK-positive multinuclear giant cells, along with the concomitant production of osteoid and new woven bone.^17^,^22^ These histopathologic changes correlated with an increase in radiographic density on computed tomography scanning.^22^ An initial planned interim analysis of the first 100 patients treated with denosumab therapy whose planned surgery was associated with severe morbidity found that 74 % had not undergone surgery for GCTB and that 16 % had a surgical procedure associated with less morbidity.^23^ At a median follow-up of 9.2 months (interquartile range [IQR] 4.2–12.9 months), 61 % of patients derived clinical benefit from denosumab, including pain reduction and improved mobility and function.^23^ In this study, we confirm and extend the results from the initial interim downstaging analysis^23^ and report detailed results from an unplanned interim analysis, performed at regulatory agency request, in 222 denosumab-treated patients with evaluable, resectable GCTB whose initially planned curative intent surgery was expected to result in severe morbidity.

## Methods

### Patients and Procedures

The study design and inclusion/exclusion criteria for this open-label, phase II study were previously reported (ClinicalTrials.gov identifier NCT00680992).^23^ Briefly, adults or skeletally mature adolescents (≥12 years of age) weighing ≥45 kg with radiologic evidence of ≥1 mature long bone, histologically confirmed GCTB, radiographically measurable active disease within 1 year before study enrollment, and Karnofsky performance status ≥50 % were enrolled. Exclusion criteria included concurrent use of alternative treatments for GCTB; known or suspected diagnosis of sarcoma, non-GCTB, giant cell–rich tumors, brown cell bone tumor of hyperparathyroidism, or Paget disease; diagnosis of a second malignancy in the past 5 years; history or current evidence of osteonecrosis or osteomyelitis of the jaw, active dental or jaw problems necessitating oral surgery, or nonhealed dental or oral surgery; or pregnancy.

Enrolled patients were separated into three cohorts.^23^ Patients from cohort 2 who were evaluable for surgical downstaging were included in this analysis. These patients had planned GCTB surgery that was associated with functional compromise or severe morbidity based on either the planned procedure, such as joint resection, limb amputation, or hemipelvectomy, or the extent or location of the lesion. The study was approved by the independent ethics committee or institutional review board for each study center. All patients provided written informed consent. The cutoff date for the data analysis was 30 August 2013.

### Procedures

Patients received open-label subcutaneous denosumab 120 mg every 4 weeks, with additional doses administered on days 8 and 15 during the first month of therapy only. For patients who had complete tumor resection, denosumab therapy continued for six additional doses after resection. In all other cases, denosumab therapy continued per protocol until either disease progression, recommendation of discontinuation by the investigator or sponsor, absence of clinical benefit according to the investigator’s judgment, withdrawal of patient consent, pregnancy, or use of any proscribed treatments. All patients were strongly advised to take daily supplements of ≥500 mg calcium and ≥400 IU vitamin D.

Curative intent surgical procedures planned at study entry were recorded prospectively, and actual surgical procedures performed after denosumab treatment were reported by investigators. Procedure selection and timing were based on serial review of radiographic imaging and clinical response by the treating physician. Disease status and clinical benefit (investigator-determined, every 4 weeks) were based on physical examination, patient report of symptoms, and serial radiologic imaging assessment per local standard practice. Serial radiographic assessments^24^,^25^ of GCTB lesions were performed per local practice guidelines, and the recommended surgical intervention was provided; the procedure was ranked using an invasiveness and postsurgical functional deficit scale.^12^,^24^ The initially recommended surgeries ranged from curettage to hemipelvectomy (invasiveness/postoperative functional impairment scale detailed in electronic supplementary Table S1).

### Safety Assessment

Adverse events (AEs) and serious AEs (SAEs) were recorded and graded according to National Cancer Institute Common Terminology Criteria for Adverse Events (CTCAE) version 3.0.23.^26^

### Statistical Analysis

Statistical analyses were descriptive in nature, and only summary statistics were presented. Efficacy and safety analyses included patients who enrolled, received at least one dose of denosumab, and were evaluable for surgical downstaging. No formal sample size calculations were undertaken. Descriptive statistics included median (IQR) as appropriate for continuous variables, and frequency (%) for categorical variables.

## Results

**Fig. 1:**
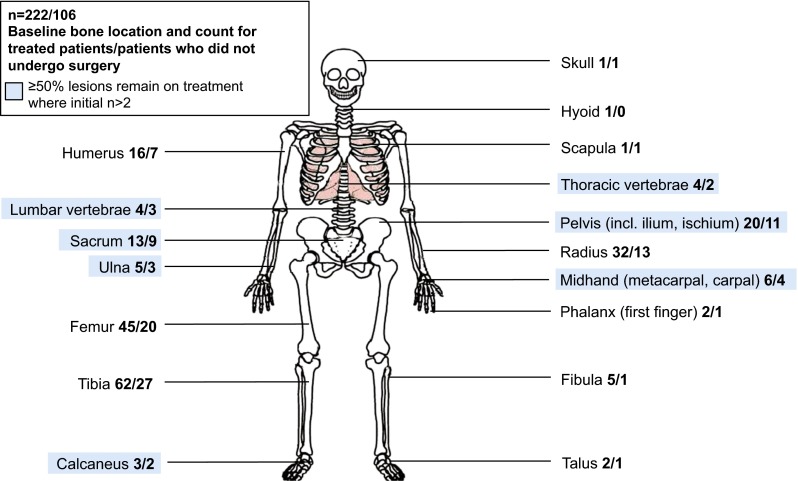
Giant cell tumor of bone lesion location at baseline and operative status. Lesion locations highlighted in *blue* show sites where ≥50 % of patients remain on denosumab without curative intent surgery

Baseline demographics and disease characteristics of patients in our cohort are shown in Table 1 and Fig. 1. Of the 222 patients enrolled and evaluable for surgical downstaging, 54.1 % (*n* = 120) were female and 80.2 % (*n* = 178) were white. The median (IQR) age was 34 (25–44) years. The lesions were in the lower (52.7 %; *n* = 117) and upper (27.9 %; *n* = 62) extremities or axial skeleton (14.9 %; *n* = 33). The majority (66.7 %; *n* = 148) of patients presented with primary GCTB, and 33.3 % (*n* = 74) of patients had a recurrent tumor following a previous curative intent surgical procedure.

**Table 1:** Baseline demographics and disease characteristics

Demographics/characteristics	Primary GCTB (*n* = 148)	Recurrent GCTB (*n* = 74)	All patients^a^ (*N* = 222)
Sex, *n* (%)			
Female	80 (54.1)	40 (54.0)	120 (54.1)
Male	68 (45.9)	34 (46.0)	102 (45.9)
Race/ethnicity, *n* (%)			
White	117 (79.1)	61 (82.4)	178 (80.2)
Asian	10 (6.8)	4 (5.4)	14 (6.3)
Hispanic	10 (6.8)	3 (4.1)	13 (5.9)
Black	8 (5.4)	4 (5.4)	12 (5.4)
Other	3 (2.0)	2 (2.7)	5 (2.3)
Age, years, median (Q1, Q3)	34 (26, 43)	35 (25, 46)	34 (25, 44)
GCTB presentation status, *n* (%)			
Primary	148	–	148 (66.7)
Recurrent	–	74	74 (33.3)
Planned surgery at presentation, *n* (%)^b^			
Hemipelvectomy	10 (6.8)	0	10 (4.5)
Amputation	21 (14.2)	17 (23.0)	38 (17.1)
Joint/prosthesis replacement	17 (11.5)	8 (10.8)	25 (11.3)
Joint resection/fusion	22 (14.9)	11 (14.9)	33 (14.9)
En bloc resection	57 (38.5)	26 (35.1)	83 (37.4)
En bloc excision	4 (2.7)	4 (5.4)	8 (3.6)
Marginal excision	1 (0.7)	0	1 (<1.0)
Curettage	9 (6.1)	8 (10.8)	17 (7.7)
Other	7 (4.7)	0	7 (3.2)

*GCTB* giant cell tumor of bone, *Q1, Q3* quartile 1, quartile 3

^a^Patients evaluable for surgical downstaging

^b^Percentages may not add up to 100 due to rounding

### Exposure and Treatment Duration

**Fig. 2:**
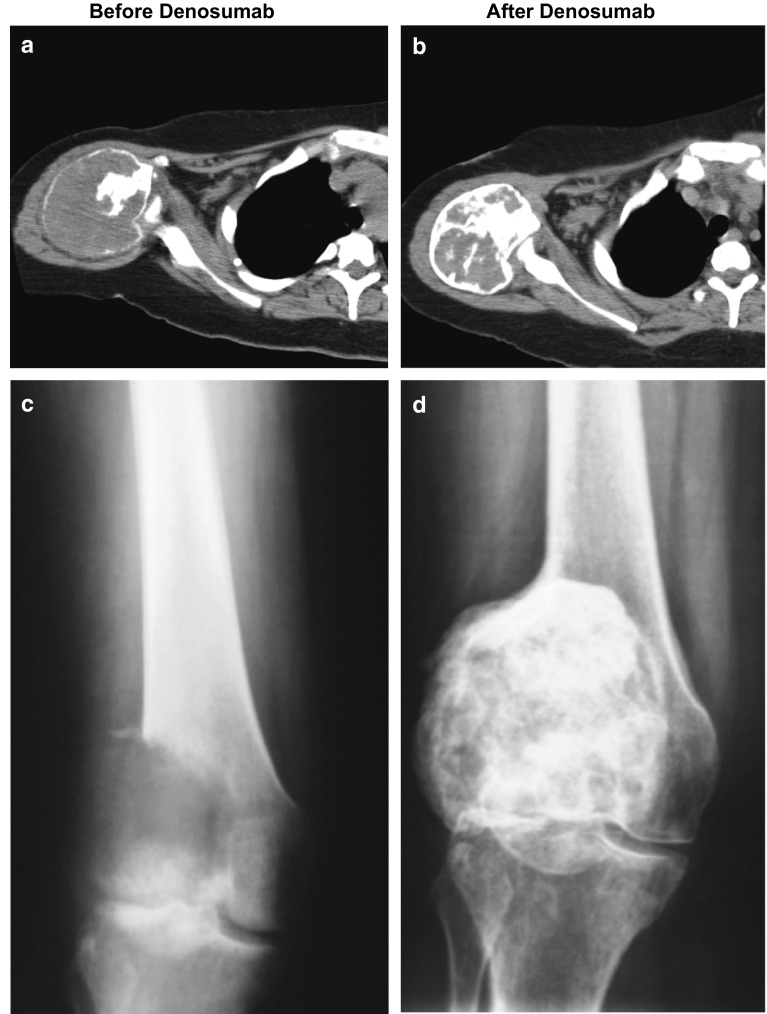
Example of radiographic images of giant cell tumor of bone of the proximal humerus and distal femur before (**a**, **c**) and after (**b**, **d**) denosumab therapy. The initial lesions were expansile with a thin peripheral calcified shell and primarily soft tissue density centrally (**a**) and showed extensive soft tissue displacement with progression following radiotherapy 2 years previously (**c**). After 4 months of treatment with denosumab, the peripheral calcification was thicker, the central lesion more heavily mineralized, and the overall size was slightly decreased (**b**, **d**)

As of the cutoff date for this analysis, the 222 patients enrolled in this surgical downstaging cohort were treated with denosumab for a median (IQR) duration of 15.3 (12.1–23.6) months. In the 106 patients who had not yet had surgery and continued on monthly denosumab per protocol, a median (IQR) of 22.5 (15.0–34.0) doses of denosumab were administered for a median of 19.5 (12.4–28.6) months (electronic supplementary Fig. S1, Panel A). In the 116 patients who underwent surgery, the median (IQR) duration of denosumab treatment was 14.2 (12.0–17.7) months (electronic supplementary Fig. S1, Panel B). Treatment with denosumab resulted in radiologic evidence of an arrest in bone lysis and the interval development of new intralesional calcification (measured as increasing density [average Hounsfield unit density] on computed tomography), increases in cortical bone thickness (including the reappearance of cortical integrity), and an overall reduction in GCTB lesion size (measured in terms of longest measured lesion diameter) [example radiographs shown in Fig. 2].

### Planned Versus Performed Surgery

In this cohort of patients, most had either not yet undergone surgery (48 %; *n* = 106/222) and remained on denosumab therapy or had undergone a less morbid procedure than originally planned (38 %; *n* = 84/222; Table 2). High morbidity procedures were avoided in 80 % of patients with either a planned hemipelvectomy (*n* = 8/10) or planned amputation (*n* = 32/40). Eighty-eight percent (*n* = 7/8) of patients with a planned en bloc excision and 37 % (*n* = 31/85) of patients with a planned en bloc resection were managed without surgical intervention in the reported follow-up period. Of the 85 patients with a planned en bloc resection, 85 % (*n* = 71) were able to have a less invasive or bone excision–sparing procedure or no surgery at all. The native joint preservation rate was 96 % (*n* = 24/25) in patients with a planned joint/prosthesis replacement and 86 % (*n* = 30/35) in patients with a planned joint resection/fusion. Of the 18 patients with planned curettage at baseline, 44 % (*n* = 8) required no surgery, 39 % (*n* = 7) underwent curettage as planned, and 17 % (*n* = 3) required en bloc resection.

**Table 2:** Planned versus actual surgery in the study cohort (*N* = 222)

Procedures associated with a higher surgical morbidity were performed in six cases on study that were not planned at study entry. There were three cases in which curettage was planned and an en bloc resection was performed: two cases with lesions located in vertebral bodies, associated with significant soft tissue extension and bony destruction involving adjoining ribs with significant spinal cord compression, and one case with a rapidly growing 7-cm mass that originated in the posterior iliac spine but displayed evidence of cortical break and had invaded the paravertebral and psoas muscles extensively. For the remaining three cases, there was one case each in which an en bloc excision was planned and an en bloc resection was performed (proximal tibia lesion that had recurred twice before referral for trial enrollment), an en bloc resection was planned and a joint/prosthesis replacement was performed (recurrent proximal tibia lesion that had been resected 13 months previously with placement of hardware), and a marginal excision was planned and an amputation performed (proximal first phalanx lesion with radiographic evidence of involvement of the articulating distal metacarpal head).

This cohort study is remarkable in that 33 % of patients who enrolled had already had one or more local recurrences after failed primary curative intent surgery. The patients with locally recurrent GCTB had very similar results to those seen in the primary GCTB population. Specifically, 45 (61 %) had not yet undergone surgery at the data cutoff date, 17 (23 %) underwent a less morbid procedure, 10 (14 %) underwent surgery as planned, and only 2 (3 %) underwent a more invasive morbid procedure. Importantly, of 17 initially recommended amputations in the locally recurrent population, none were required to date (see outcomes in patients following local recurrence in electronic supplementary Table S2).

Median (IQR) duration of postoperative follow-up for all patients (*n* = 116) who underwent curative intent surgery was 13.0 (8.5–17.9) months. Local recurrence was reported in 15 % (*n* = 17/116) of patients who had surgery. The median duration of postoperative time until recurrence in the 17 patients who experienced local recurrence was 13.6 (10.5–15.7) months. In the 99 patients who underwent surgery but had not experienced recurrence by the time of data cutoff, the median postoperative follow-up time was 12.9 (7.8–18.0) months (see electronic supplementary Fig. S2). Of the 17 patients with local recurrence following denosumab therapy, 14 underwent curettage as their initial on-study GCTB surgery, 2 underwent en bloc resection, and 1 had a joint resection. The median number of doses of denosumab given in the adjuvant setting per protocol was 6.0 (IQR 3.0–6.0). Local recurrence was reported in 5 of the 29 patients (17 %) with recurrent GCTB at enrollment who underwent surgery after on-study treatment with denosumab.

### Adverse Events

AEs of any grade occurring with >10 % frequency were as follows: arthralgia (24.8 %), fatigue (20.7 %), pain in extremity (19.4 %), headache (18.9 %), nausea (18.0 %), and back pain (10.8 %). Grade 3 or 4 AEs occurring with a ≥1 % frequency were hypophosphatemia (2.7 %) and pain in extremity (1.4 %). Twenty-one (9.5 %) patients experienced SAEs, and nine (4.1 %) experienced AEs that resulted in treatment discontinuation (Table 3). Of the 21 SAEs reported by investigators, only two occurred more frequently than once (appendicitis and osteitis; both *n* = 2, 0.9 %). There was one case each of osteonecrosis, nondisplaced tibia fracture, back pain, other neoplasm, and myeloproliferative disorder. Four (1.8 %) patients were reported with malignant GCTB transformation on study: two within-field, radiation-associated sarcomatous transformations at 4 and 6 years after radiotherapy, respectively, and two with pelvic or sacral GCTB lesions that progressed on denosumab by 257 days of exposure. In each of these latter two cases, a diagnosis of primary malignant GCTB was felt by the investigator to have been missed by sampling error at initial core biopsy. Nonserious occurrences of hypocalcemia were observed in 3.2 % of patients; no serious occurrences were reported. Only one patient reported osteonecrosis of the jaw (CTCAE grade 1), which resolved by the data cutoff date.

**Table 3:** Patients with adverse events^a^

Patients with AEs	Study cohort (*N* = 222) [*n* (%)]
Overall safety summary	193 (86.9)
AEs occurring with >10 % frequency	
Arthralgia	55 (24.8)
Fatigue	46 (20.7)
Pain in extremity	43 (19.4)
Headache	42 (18.9)
Nausea	40 (18.0)
Back pain	24 (10.8)
Grade 3 or 4 AEs	33 (14.9)
Hypophosphatemia^b^	6 (2.7)
Pain in extremity^b^	3 (1.4)
Serious AEs	21 (9.5)
AEs leading to treatment discontinuation	9 (4.1)
AEs of interest	
Hypocalcemia (nonserious)	7 (3.2)
Serious infections	6 (2.7)
Adjudicated positive osteonecrosis of the jaw^c^	1 (<1)

*AE* adverse event

^a^Based on Medical Dictionary for Regulatory Activities, version 14.1, and Common Terminology Criteria for Adverse Events, version 3.0

^b^Hypophosphatemia and pain in extremity were the only grade 3 or 4 AEs occurring with a frequency ≥1 %

^c^One case of osteonecrosis of the jaw resolved by the cutoff date

## Discussion

Among patients with resectable GCTB treated with denosumab and for whom curative intent surgery was planned and believed to be associated with significant morbidity before enrollment, 48 % had not yet undergone surgery altogether and remained on monthly denosumab treatments at the time of the data cutoff. Another 38 % of patients were treated with denosumab and underwent a less invasive surgical procedure than was planned at study entry. The patients who underwent a curative intent procedure while on study have not yet experienced an increased local recurrence rate (15 %, at a median postoperative follow-up of 13.0 months for the 116 patients who underwent surgery) or rebound effect following discontinuation of denosumab treatment. These results support the conclusion that denosumab therapy may represent an important option for patients with resectable GCTB to avoid immediate surgery, control disease, or achieve equivalent surgical outcomes with less morbid procedures.

For patients with resectable GCTB tumors, disease control can be achieved with wide surgical excision or less invasive intralesional curettage. GCTB is usually surgically treated with intralesional curettage combined with high-speed burring, which is the least invasive surgical option, improving the thoroughness of tumor removal and allowing preservation of the joint adjacent to the tumor. Recurrence rates associated with intralesional curettage using bone graft as void filler and no additional adjuvants (such as cryotherapy or phenol) are reported to be between 12 and 65 %.^25^,^27^–^33^ Although wide excision is associated with a lower risk of local recurrence (up to 12 %),^25^,^27^,^28^,^30^,^34^ it is necessarily associated with poorer long-term functional consequences due to greater bone loss and the limitation of joint motion due to resection reconstruction. In view of these risks, deferring surgery or downstaging the surgical procedure needed to treat GCTB may offer substantial clinical benefits to patients.

Denosumab may permit less invasive procedures for patients with GCTB without deleterious outcomes, possibly serving as a contrast to previous reports indicating that highly morbid procedures are required to limit disease progression and recurrence.^25^,^33^,^35^,^36^ The native joint preservation was >85 % in patients with planned joint/prosthesis replacement or joint resection/fusion with denosumab treatment. In addition, even in cases where prosthetic replacement was performed, reduction in the size of the tumor mass and bone healing around the periphery of the tumor can facilitate complete en bloc tumor resection. Furthermore, there are several patients in the study in whom highly invasive surgery (e.g. amputation, hemipelvectomy, or axial skeleton surgery) was planned who remained on treatment with denosumab after achieving disease control, thus far without the need for highly invasive surgical intervention.

Recurrence rates in this study following surgical resection were similar to published experience (between 12 and 65 %^25^,^27^–^33^), which is particularly notable given the location of the tumors in our cohort, as well as the number of patients (*n* = 74; 33.3 %) with recurrent disease. These findings suggest that downstaging of the surgical invasiveness in patients treated with denosumab has not given rise to an increase in recurrence rate at a median postoperative follow-up of 13.0 months. Although these data must be interpreted with some caution given the follow-up time, previous collaborative group studies^34^ and longitudinal institutional case series^9^,^31^ have shown that local recurrence following surgery tends to occur predominantly within the first postoperative 12–18 months. No new safety risks were observed in this population of patients with GCTB receiving denosumab therapy. Osteonecrosis of the jaw, as well as hypocalcemia, were observed at low rates, consistent with previous studies of denosumab.^23^ Additional protocol-directed follow-up time of these patients (for 60 months total following surgery) will continue to reveal whether surgical downstaging modifies the long-term risk of postsurgical local recurrences in this population.

We report six cases in which procedures associated with a higher surgical morbidity that were not planned at study entry were performed on study. In each of these cases, the patient experienced radiographic response (defined as a reduction in size and/or increase in calcification), clinical benefit (defined as a reduction in pain and/or improvement in function or mobility), or both. Aside from a grade 3 wound infection in the patient who underwent resection of his iliac lesion, there were no reported intraoperative or postoperative surgical complications, and none of these six patients had experienced local or distant recurrence at the time of the data cutoff.

## Conclusions

As of the cutoff date for this interim analysis, treatment with denosumab decreased the need for surgical intervention and reduced surgical morbidity in patients with GCTB who underwent surgery with curative intent. These findings support the use of denosumab in a preoperative setting to defer or downstage the planned surgical procedure in patients with GCTB when surgical resection is likely to result in severe morbidity.

## Electronic supplementary material

Supplementary material 1 (DOCX 262 kb)
