# Supplementary material for: Surgical Downstaging in an Open-Label Phase II Trial of Denosumab in Patients with Giant Cell Tumor of Bone
Source: Ann Surg Oncol. 2015 Jun 2;22(9):2860–8. doi: 10.1245/s10434-015-4634-9 (PMC4531146; doi:10.1245/s10434-015-4634-9)
Supplement: Supplementary file 1 — Supplementary material 1 (DOCX 262 kb) [file 10434_2015_4634_MOESM1_ESM.docx]

**Supplementary Material**

**Supplementary Table S1. Criteria for Assessment of Recommended Surgical Procedure^12,24^**

| **Category** | **Procedure** | **Description** |
| --- | --- | --- |
| 0 | Surgery not required | N/A |
| 1 | Curettage | Intralesional procedure involving visible removal of tissue considered grossly as tumor; may include use of burring, adjuvants, or bone allografting |
| 2 | Marginal excision | Lesion removed in one piece; plane of dissection is through the pseudocapsule or reactive tissue around the lesion |
|  | En bloc excision | Intracompartmental resection en bloc, with cuff of normal tissue (wide excision) |
|  | En bloc resection | Radical resection, en bloc, of the lesion, pseudocapsule, reactive zone, and entire bone or muscle |
| 3 | Joint resection | Resection of complete joint |
|  | Joint resection with prosthesis | Resection of complete joint with placement of a prosthesis |
| 4 | Amputation | Removal of entire involved bone |
| 5 | Hemipelvectomy | Removal of an entire leg together with the lateral half of the pelvis on the same side |
| 6 | Unresectable | Surgery with curative intent not possible |

NA=not applicable.

**Supplementary Table S2. Planned Versus Actual Procedures for Patients Presenting With Recurrent GCTB (n=74)**

|  | Actual On-Study Procedure, n (%) | | | | | | | | |
| --- | --- | --- | --- | --- | --- | --- | --- | --- | --- |
| Planned Procedure | No Surgery **(n=45)** | Curettage **(n=17)** | Marginal Excision **(n=2)** | En Bloc Excision **(n=1)** | En Bloc Resection **(n=5)** | Joint Resection/ Fusion **(n=2)** | Joint/Prosthesis Replacement **(n=2)** | Amputation(n=0) |  |
| Amputation (n=17) | 14 (82) | – | 2 (12) | – | – | – | 1 (6) | – |  |
| Joint/prosthesis replacement (n=8) | 5 (63) | 2 (25) | – | 1 (13) | – | – | – | – |  |
| Joint resection/ fusion  (n=11) | 7 (64) | 1 (9) | – | – | 1 (9) | 2 (18) | – | – |  |
| En bloc resection (n=26) | 13 (50) | 9 (35) | – | – | 3 (12) | – | 1 (4) | – |  |
| En bloc excision (n=4) | 3 (75) | – | – | – | 1 (25) | – | – | – |  |
| Marginal excision (n=0) | – | – | – | – | – | – | – | – |  |
| Curettage (n=8) | 3 (38) | 5 (63) | – | – | – | – | – | – |  |
|  | No surgery n=45 | | Less morbid n=17 | | Planned n=10 | | More morbid n=2 | |  |

GCTB=giant cell tumor of bone.**Supplementary Figure S1.** Exposure duration of patients treated with denosumab, including (A) patients who had not yet undergone surgery and (B) patients who underwent surgery on study.

**Supplementary Figure S2.** Postoperative follow-up time for all patients in the study cohort who underwent curative intent surgery on study. (A) Red lines represent the duration of postoperative follow-up in months for patients without recurrence. (B) Blue lines represent the interval of time in months between curative intent surgery and relapse in patients who experienced recurrence.
